# Electron microscopy: essentials for viral structure, morphogenesis and rapid diagnosis

**DOI:** 10.1007/s11427-013-4476-2

**Published:** 2013-05-01

**Authors:** Ying Zhang, Tao Hung, JingDong Song, JinSheng He

**Affiliations:** 1 College of Life Sciences and Bioengineering, Electron Microscopy Laboratory, School of Science, Beijing Jiaotong University, Beijing, 100044 China; 2 Institute for Viral Disease Control and Prevention, Chinese Center for Disease Control and Prevention, Beijing, 100052 China

**Keywords:** electron microscopy, viral structure, viral morphology, viral diagnosis

## Abstract

Electron microscopy (EM) should be used in the front line for detection of agents in emergencies and bioterrorism, on accounts of its speed and accuracy. However, the number of EM diagnostic laboratories has decreased considerably and an increasing number of people encounter difficulties with EM results. Therefore, the research on viral structure and morphologyant in EM diagnostic practice. EM has several technological advantages, and should be a fundamental tool in clinical diagnosis of viruses, particularly when agents are unknown or unsuspected. In this article, we review the historical contribution of EM to virology, and its use in virus differentiation, localization of specific virus antigens, virus-cell interaction, and viral morphogenesis. It is essential that EM investigations are based on clinical and comprehensive pathogenesis data from light or confocal microscopy. Furthermore, avoidance of artifacts or false results is necessary to exploit fully the advantages while minimizing its limitations.

Electron microscopy (EM) has had a profound impact on our knowledge and understanding of viruses and infectious diseases [1,2], although this technique is occasionally considered to be laborious, old-fashioned and unnecessary [3,4]. As a result of its rapid turnaround time and ability to detect unknown and unsuspected organisms, EM is on the front line in surveillance of new outbreaks [1,5–7]. Viruses from various families exhibit distinct appearances and these morphological variations form the basis of virus identification by EM [8,9]. EM allows visualization of the structural characteristics [10] and morphogenesis [11] of viruses from a variety of clinical specimens. This information is often sufficient for identification of unknown infectious agents in the clinic.

The first electron microscope was built in 1931 by Ernst Ruska with the help of his mentor Max Knoll, as part of his Ph.D. studies [5,12]. Eight years later, Ruska and his colleagues, Kausche and Pfankuch, were the first to visualize viruses (*Tobacco mosaic virus*) using EM [12], and in 1986, Ruska shared the Nobel Prize with Binnig and Rohr, developers of the scanning tunneling electron microscope. EM has a history of about 100 years and is used routinely to detect and characterize morphologically all structures greater than 15 nm in diameter, which fortunately coincides with the size of the smallest viruses. Transmission electron microscopy (TEM) and scanning electron microscopy (SEM) are used to investigate internal and surface structures, respectively. However, conventional TEM is used for virus detection in preference to SEM. TEM visualizes both external and internal virus structures as 2D information, whereas SEM data are confined to surface topology presented in 3D [4]. Ultramicro-detection, dynamic observation and quantitative measurement can be performed by EM, based on the high resolution and various sample preparation methods. Besides analysis of viral structure, EM provides a technique for investigation of viral morphogenesis. Here, we review the technological advantages, historical contribution to virology, limitations of EM, and direction in future studies of viral ultrastructure, morphology and morphogenesis.

## 1 Technological advantages

The majority of direct and indirect virus detection techniques, including ELISA, immunohistochemistry, *in situ* hybridization, polymerase chain reaction (PCR), virus titration and plaque formation, are tailored to detect a single species or family of agents with high sensitivity and specificity. However, EM has still served in qualitative and quantitative analysis rather than general sample observation. EM resolution of structures has been defined at a very high level: (i) titers of $10^5$–$10^6$ particles $mL^{-1}$; (ii) spot space of 0.1 nm; (iii) ultramicro-quantities of $10^{-25}$ g [13]. Based on traditional EM, functional EM is used for immuno-negative staining, immuno-EM (IEM) [14–16], ion trapping, element analysis, cryo-EM and 3D reconstruction; all of which play important roles in studies of viral structure and morphogenesis [9,13,17]. In this review, we focus on EM sensitivity in viral structure and morphology-based detection instead of comparison with other molecular method [18].

### 1.1 Direct and "open view"

The main area in which EM has been used in diagnostic virology is the evaluation of stool specimens from patients with suspected viral gastroenteritis. None of the major viral causes, including rotaviruses, enteric strains of adenovirus, noroviruses, and astroviruses, are easily cultured. All can be seen by TEM of material that has been negatively stained using phosphotungstic acid (PTA) or uranyl acetate (UA). Other applications of EM include direct examination of blister fluid to discriminate between *herpesvirus* and *poxvirus* particles and detection of *filoviruses* (e.g., *Marburg* and *Ebola viruses*) in clinical samples such as serum or urine [14].

Viruses vary in size from 20 nm for enteroviruses to large pleomorphic myxoviruses, which can exceed 1 μm in length. Cuboidal, hexagonal and complex symmetry form the main structural types of viruses [13,19]. Structural features, such as the envelope with glycoprotein spikes on the surface (not all viruses with cuboidal symmetry have an envelope), the capsid, the capsomere and core containing nucleic acids in cuboidal viruses or glycoprotein spikes, matrix proteins, envelope and ribonucleoproteins in helical viruses can be discerned clearly by TEM through negative staining by different methods. Based on the principle of the "open view" (a term coined by Hans Gelderblom) [3], TEM can be used to analyze: (i) integrity of viral particles; (ii) appearance of viral envelopes; and (iii) distribution of viruses in organs, tissues and cells. EM is also used to detect multiple infections and even infections that have not been screened for because they were considered unlikely to occur. The use of negative staining in EM in emergency situations caused by infectious agents is essential to characterize many new isolates detected in diagnostic cell cultures and clinical samples including stools, urine, and biopsy specimens [3,20]. Therefore, the combination of morphological and structural information has forms the fundamental criteria for initial classification of many agents. Currently, more than 30 000 different viruses comprising 56 separate families have been identified, and humans have been found to host 21 of the 26 families specific for vertebrates [21].

### 1.2 Rapidity and Specificity

If available and performed skillfully, EM diagnosis can usually be completed within 15 min and confirmed within 1 h by immune-negative staining with specific antibodies or serum. Microwave-assisted tissue processing for one-day EM diagnosis, exactly 4–5 h, provides rapid detection and allows a significant reduction in sample preparation time from days to hours, without any loss in ultrastructural detail [22]. Recognition of structural features and proteins defined as diagnostic markers and of patterns in size and particle morphology leads to rapid identification of infectious agents [4].

Virus localization and virus cell interactions can be traced by IEM, Enzyme IEM (EIEM) or ultrathin sectioning. The specificity of IEM is based on antibody virus reactions, which were first observed in 1941 with *Tobacco mosaic virus* [23,24]. In this instance, the term "immune EM" was coined by Almeida and Waterson [25] to describe the clumping of viral particles in EM. IEM usually involves two main techniques: immunoaggregation and immunolocalization. Immunoaggregation is an effective method for virus enrichment, which is used in solid phase IEM to increase the sensitivity of virus detection through cross linked antibody-mediated viral aggregation if the virus concentration is too low to be seen easily. The sensitivity of EM is increased 100-fold if homologous antibody is used to aggregate the virus [17]. Visualization of virus-antibody aggregates forms the basis for serotyping by IEM. Aggregation of viruses by antibodies has also been used to identify viruses specifically and to attract them to grids [20]. Immunolocalization can be used to identify many viral proteins, engineered viral proteins, virus-like particles and whole viruses. Studies with viral antibodies and gold-labeled secondary antibodies have demonstrated the intracellular location of various viral proteins, which sheds light on how these proteins are assembled. Antibodies against engineered proteins can be used to demonstrate differences by comparison with the native proteins, or to confirm the presence of artificial virus-like particles. Two types of procedures are available for specific identification of whole viruses: ultrathin sections stained with primary antibody followed by secondary antibody conjugated with colloidal gold, or immunolabeling combined with negative staining. Immunolabeling of hantavirus-infected cells has been used to show that the inclusion bodies (IBs) were composed of nucleocapsid proteins, despite their appearance in various forms (polygon, bunched, filiform or super-large) [20,26].

### 1.3 Accuracy and high resolution

EM has been recognized as a high-resolution (0.1 nm) nano-science and nanotechnology. Acceleration of monochromatic electrons to 80–100 kV provides clear visualization of specimens with a thickness of up to 100 nm [4] and magnification of many thousands of times. This allows the fine detail of viruses to be discerned at the nanometer scale. Furthermore, viral particles and their constituents are visualized in different forms by using negative staining and thin sectioning. Negative staining has the advantage of providing a high-resolution outline of viral surface structures, such as the capsid and its capsomeres or the lipid membrane. In contrast, thin sections provide structural information about both outer and inner constituents, such as a second capsid, the core, and ribonucleoproteins, especially in cell culture samples.

Viral size, shape, and substructure differ significantly among the different families. Under EM, viruses with a range of sizes can be accurately identified based on structural features, such as the regular protein shell comprising multiple copies of one or more proteins forming the capsid of parvo and piconarviruses and the additional lipid membrane studded with glycoprotein projections of much larger viruses. Determination of the size of a suspicious particle, its capsid symmetry, the presence of additional structural components (e.g., glycoprotein projections and lipid membranes) ultimately leads to assignment of the viral particle to a particular family. For instance, identification of icosahedral capsid symmetry (70–80 nm in diameter) with surface capsomeres represents the distinct structural features for diagnosis of adenovirus.

## 2 Application of EM in clinical and basic virology

### 2.1 Historical contribution of EM to the discovery of new viruses

Since the first electron micrograph of poxvirus was published in 1938, EM has played an important role in the discovery of new viruses and diagnosis of emerging infectious diseases with unknown etiology [1,2,8,27,28]. Within the subsequent 30 years, EM has continuously contributed to the initial identification of many other viral agents, including the differential diagnosis of smallpox and chicken pox infections in the late 1940s, and the discovery of poliovirus in 1952 [29] and many gastrointestinal viruses, including hepatitis B virus (HBV) [30], reoviruses [28] and enteroviruses in the late 1960s [31,32]. In the past 40 years, more than 40 novel pathogens have been described, half of which are viruses, such as Ebola virus, hantavirus, hepatitis virus and severe acute respiratory syndrome (SARS) coronavirus (Table 1). Most of these etiological studies depended significantly on morphological visualization by EM, and the viruses detected were often determined to be the cause of severe or unusual in patients. EM was instrumental in elucidating the viral agent responsible for the first public health emergency and furthermore, this technique continues to be used to confirm infection and in quality control of molecular techniques [42].

Most importantly, one of the main advantages of using EM for viral diagnosis is that it does not require organism-specific reagents for recognizing the pathogenic agent. Other approaches involving molecular and serological methods require the availability of a specific probe for viral identification. In the event of a disease caused by an unknown pathogen, it is hard to know which reagent to select for identification. In the 1980s, two world-famous discoveries of new viruses occurred in China: adult diarrhea rotavirus (ADRV) group B and hemorrhagic fever with renal syndrome (HFRS) virus. It was through EM and IEM that a new type of Rotavirus was identified and the causative agent of HFRS was classified [20,37]. HFRS virus particles are morphologically similar in EM analysis to members of Bunyaviridae, such as Hazara, Chagres, Congo, Ganjam and sandfly fever viruses. The three main morphological components observed in HFRS virions are [15] (i) distinct unit membrane (i.e., the envelope); (ii) a granulo-filamentous viroplasm enclosed inside the membrane; and (iii) visualization of short projections on the membrane, which are not usually apparent due to immune precipitates. Therefore, it has been concluded that viruses isolated in China that are responsible for classical and mild forms of HFRS appear to possess morphology and morphogenesis essentially similar to bunyavirus-like viruses. Hung et al. [37,44] have confirmed EM detection and classification of ADRV on the basis of morphology by RNA profile analysis. Two epidemics of acute diarrhea in China in late 1982/early 1983 affected more than 12000 adults in two coal-mining districts. The virus isolated from stool samples resembled a rotavirus morphologically. The viral particles observed in thin sections were indistinguishable from ordinary rotaviruses, exhibiting a dense hexagonal core surrounded by a characteristic radiating spoke-like structure. The mean diameter of

**Table 1** Historical contributions of EM and IEM to new virus outbreaks

| Year | Outbreaks of unknown etiology | References |
|---|---|---|
| 1970 | Dane particles in HBV | Dane et al. (EM) [33] |
| 1971 | Norwalk | A. Z. Kapikian [31] |
| 1973 | HAV | S. M. Feinstone, A. Z. Karpikian et al. [34] |
| 1973 | Infantile diarrhea (Group A) | R. Bishop et al. [35] |
| 1976 | Ebola in Zaire | F. Murphy (EM) [36] |
| 1983 | Hemorrhagic fever with renal syndrome (HFRS) in Mainland China | T. Hung et al. (EM+IEM) [20] |
| 1983 | Adult Diarrhea Rotavirus (ADRV, Group B) in Mainland China | T. Hung et al. (EM+IEM) [37] |
| 1995 | Hantavirus pulmonary syndrome (HPS) | S. Zaki (PCR+ IEM) [38,39] |
| 1996 | Henipavirus (Hendra and Nipah) in Australia and Asia | [40,41] |
| 2003 | Severe acute respiratory syndrome (SARS) coronavirus | J. S. M. Peiris, K. Y. Yuen et al. (PCR) [6,42] |
| 2003 | Monkeypox in USA | [43] |

the particles was 70 nm. In negative staining, particles from the fecal supernatants also showed typical rotaviral morphology. Unlike those in thin sections, however, particles in supernatants were frequently degraded to smooth spheres of approximately 50 nm in diameter [44].

However, a change in use has been seen over the history of diagnostic EM in virology. Starting in the 1990s and coincident with the broad introduction of modern diagnostic techniques, the number of EM diagnostic laboratories has decreased considerably, despite the obvious advantages of this technique. Molecular techniques, which offer greater sensitivity and often the capacity to process large numbers of samples easily, have replaced EM in many areas of diagnostic virology [3]. Hence, the role of EM in clinical virology is evolving with less emphasis on diagnosis and more on basic research, although this is likely only to be undertaken in specialist centers. To guarantee the performance of diagnostic EM in the future, the system of External Quality Assessment on EM virus diagnosis (EQA-EMV) was established in 1994 at the Robert Koch Institute (RKI) in Berlin. A basic laboratory course has been held annually at the RKI focusing on teaching practical techniques and skills and implementation of EM techniques in correct diagnosis, surveillance, and control of infectious diseases. The growing number of participants from many countries indicates a requirement for and an interest in this program. Today, EM still offers tremendous advantages to the microbiologist, both in terms of the speed of diagnosis and the potential for detection, using a single test, of any viral pathogen or even multiple pathogens present within a single sample [3].

## 2.2 Virus differentiation

Viral appearance varies in different samples, which make it necessary to differentiate viruses according to features visible under EM [1,45–47]. The criteria for differentiation include the following [13]. (i) Size. In negative staining, naked human viruses have three size ranges: 22–35 nm (e.g., parvoviruses, enteroviruses and caliciviruses); 40–55 nm (polyomaviruses and papillomaviruses); and 70–90 nm (reoviruses, rotaviruses and adenoviruses). Size is determined under the microscope by comparing the particle to a marker on the screen that represents a known size at a given magnification. (ii) Outer envelope. Some enveloped viruses have clearly visible surface projections (e.g., orthomyxoviruses, paramyxoviruses and coronaviruses), whereas others have short or fragile projections that are rarely seen in negative stains of clinical material (e.g., rubella virus, herpesviruses, and retroviruses). (iii) Location. The envelope in enveloped DNA viruses is formed by budding from the nuclear membrane or by movement to the cytoplasm and budding into cytoplasmic vesicles or from the plasma membrane. Naked DNA viruses may be seen in the cytoplasm after the nucleus starts to degrade. RNA viruses are not found in the nucleus with one exception; occasionally, helical paramyxovirus nucleocapsids (but not whole enveloped virions) are seen in the nucleus. The outer membrane in enveloped RNA viruses is derived by budding into cytoplasmic vesicles or from the plasma membrane. (iv) Structural features [48,49] and generality of morphogenesis. Besides structural units in the viral surface such as capsomeres and spikes, 3D structure may provide information on fine features and viral proteins [50]. The crystal structure of nucleocapsid proteins has revealed a potential therapeutic target in Crimean Congo hemorrhagic fever virus (CCHFV). The structure reveals surfaces that are likely involved in RNA binding and oligomerization, and functionally critical residues within these domains have been identified using a minigenome system that can recapitulate CCHFV-specific RNA synthesis in cells.

Differential EM diagnosis contributed to identification of the causative agent in two similar viral diseases (smallpox and chicken pox) in 1948 [27,51]. Leukovirus and human immunodeficiency virus (HIV) are similar in size and shape, but differ obviously in appearance in thin sections. Specifically, heavily stained, eccentrically located core-like structures are present in HIV, whereas leukovirus has typical C particles with a centrally located large core that is stained more heavily than surrounding structures. In negative stain-

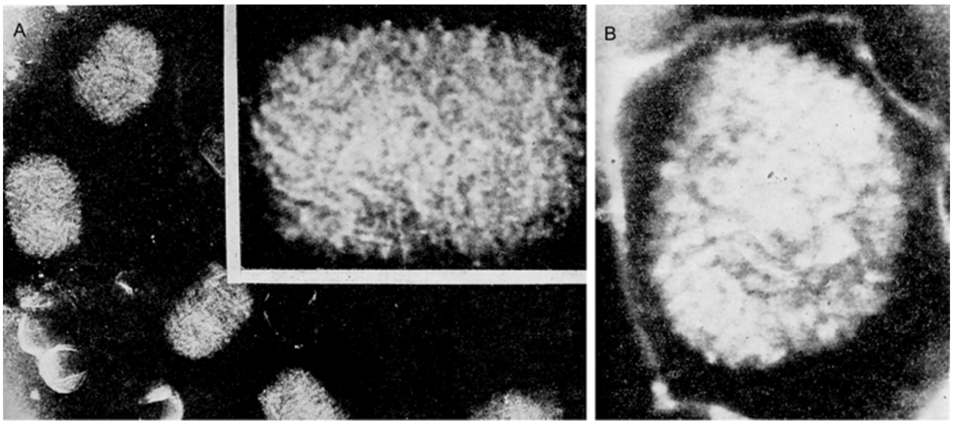

**Figure 1** Morphological differences between molluscum contagiosum virus and poxvirus [52]. Poxviruses are enveloped, whereas the particles of molluscum contagiosum are naked. Negative staining. Magnification, 80000×. (Reprinted with permission of Science Press in Beijing, China).

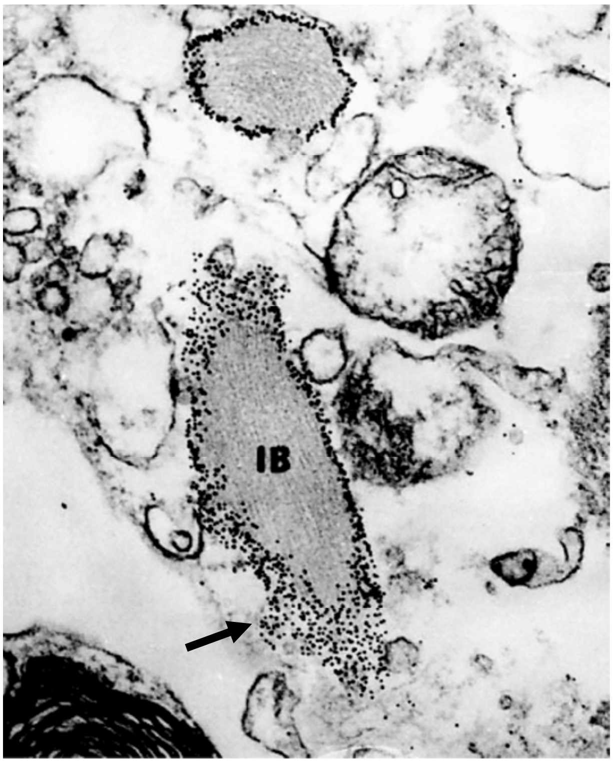

**Figure 2** Immunogold-labeled IBs in HRFS virus [15]. The filamentous IBs are specifically coupled to gold particles (arrow). Thin section, 55000×. (The EM picture was taken by Dr. Hung Tao, and reprinted with permission of Science Press in Beijing, China).

ing, molluscum contagiosum virus and poxvirus appear similar in size, shape and surface structural features but are distinguishable by the presence of an envelope in the poxvirus whereas molluscum contagiosum virus is naked (Figure 1) [52]. Furthermore, it is necessary to differentiate virus with other microbiology [53].

## 2.3 Localization of specific viral antigens

Localization and labeling of virus-specific antigens can be performed by IEM [46]. Viral appearance varies at different stages, with atypical viral particles, premature virus, mature virus, and degraded virus visualized in thin sections of tissue or cell culture samples. IEM is performed using a general procedure including antigen binding through primary antibody and an immuno-gold labeled secondary antibody. IEM has the advantage that this method can be used to determine whether atypical structures are viral components. For instance, the irregular shape of viral IBs makes diagnosis difficult. IEM provides a practical method that allows clear visualization of IB structure (Figure 2) [15,20].

A critical skill in IEM involves conservation of antigen quality in samples after complex fixation protocols and other procedures. Therefore, processes detrimental to antigen quality should be avoided and pre-fixation labeling and post-fixation techniques have been developed that are commonly used in IEM to preserve antigen information.

Double labeling can be performed using two sizes of immuno-gold particles (5 and 10 nm). This approach can be used to distinguish the similarity in ultrastructure of amyloid fibers of prion proteins [54,55,14] and β amyloid (Aβ) in Alzheimer's disease. The prion rods are indistinguishable from many other purified amyloids [55] such as Aβ [56], whereas conformation-selective antibody [57–59] based IEM can be used to recognize one type of amyloid from the others.

## 2.4 Micro analytical and quantitative EM

Quantitative EM requires the use of a hydrophilic support film to ensure even sample structure and staining. A freshly carbon-coated grid with a support film of Formvar (Collodion, Butvar or Pioloform are alternatives) provides even spreading, but older grids may not. Interactions between the virus and the grid surface as well as the stain occur in negative staining and these influence particle amount and viral structures (by fattening or shrinking), as well as leading to osmotic disruption of particles (pre-fixation or on-grid fixation of the specimen by 0.1% (v/v) glutaraldehyde can improve preservation, in particular with purified viral particles) [4]. Therefore, EM-based quantitative data should be collected and assessed with particular care. An internally controlled solution for determining particle size is provided by the addition of an internal marker with known dimensions, such as catalase crystals (lattice spacing 8.75 nm). By measuring both the virus and the marker (on the same negative using a 10× measuring magnifier) and comparing the marker values with known standard values, viral dimensions can be determined with a deviation of <5% [4]. The same principle underlying the use of an internal marker is applicable to particle enumeration. After mixing defined volumes of the viral suspension and a suspension of latex beads (or polypropylene latex particles) of known concentration, the respective particles can be counted after negative staining or thin-section TEM. The concentration of the virus is then assessed by comparison with the concentration of the latex beads [4].

## 2.5 Virus cell interaction and viral morphogenesis

Viral replication is impossible without cells, which make it important to study virus cell interactions [19,60,61] and viral morphogenesis by EM. The common life cycle of viruses comprises adhesion [62], virus host receptor binding [62], entry [63,64] or fusion [65], endocytosis [66], replication [67], assembly [68] and budding [69–71] (Figures 3 and 4). New viral particles infect other cells and start a new life cycle. Changes in cell signal transduction [72,73], apoptosis and autophagy [74] are all clues to virus infection. Therefore, the more confusing aspects of the morphology of virus cell interactions [75–77] will be deciphered on the basis of experiments in the fields of molecular biology, immunology, and virology. In the early 1980s in China, the causative agent in outbreaks of HFRS was identified by EM of viral morphogenesis. Formation of immuno-enzyme-positive IBs was the remarkable feature observed in the HFRS-virus-infected cells, but the IBs are usually atypical in the early stages of virus infection. These IBs varied in number and appearance and could only be visualized in cells processed by immunostaining. The matrix of the IBs often appeared homogenous and/or granulo-filamentous, and occasionally, profiles of incomplete particles seemed discernible inside the IBs. Some atypical, possibly immature virions were frequently seen in the vicinity of the IBs [20,26]. Hung et al. also reported their attempts in seeking Hantaan virus or its antigens, by means of thin-section IEM

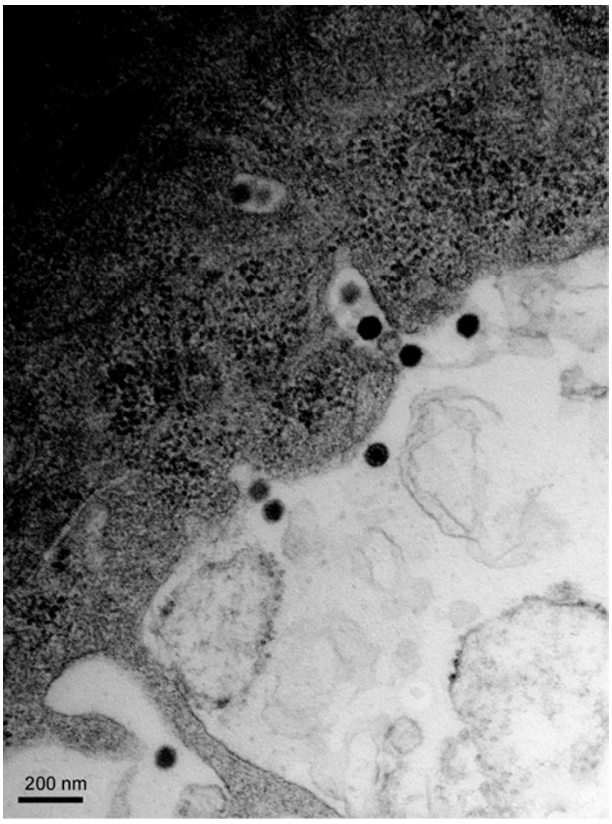

**Figure 3** Example of virus entry. Thin section: scale bar, 200 nm.

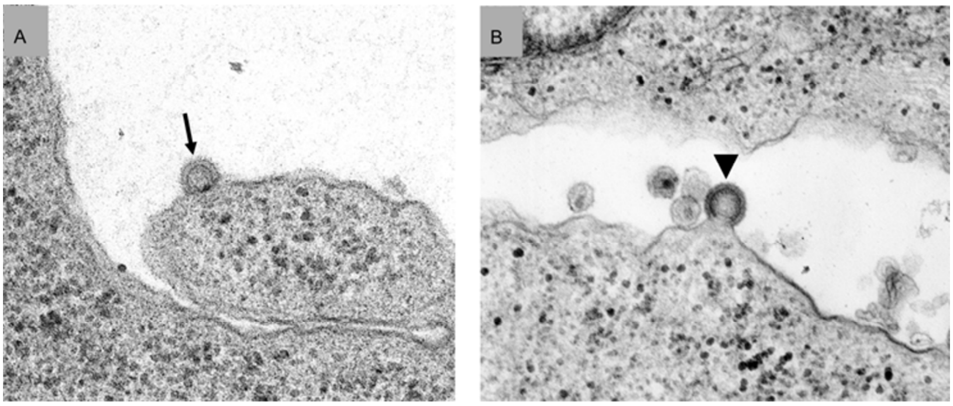

**Figure 4** Example of virus budding. HIV virus budding at the plasma membrane in different phases: (A) a tiny virus is appearing (arrow); (B) virus is falling off (arrow head). Thin section, 31000× in A and 38000× in B.

in organs of patients who died during the acute phase of HFRS. Strikingly, many dense precipitates, Hantaan virion-like structures, typical filamentous IBs, and immuno-gold-labeled viruses, as well as a surface antigen layer were observed in cadaveric kidneys. These findings may have elucidated the pathogenesis of HFRS in target organs most affected by the disease, such as the kidneys [15,20,78].

Interpretation of differences in density, shape and contrast in EM images can be daunting. However, many aspects of such images that can be used to interpret cell virus interactions are based on the results of negative staining, thin sections, EIEM and immuno-gold-labeled IEM. Studies of virus host relationships began in the mid-1950s [79,80]. Viruses were shown to vary in appearance during different stages of infections: HIV budding from infected lymphocytes, C particles of leukovirus, stick or tadpole-like HBV virus, arrays of HBV core antigens, silk, curling or foam-like influenza virus and strings of beads, kidney- or dumbbell-like SARS coronavirus [6].

Besides clinical diagnostic uses, EM is important for the study of the ultrastructural features of virus cell interactions, which in turn, are useful in elucidating the potential of antiviral agents [81]. Surface proteins on viruses are responsible for their attachment to and entry into cells [82] as well as for their ability to elicit an immune response [83].

## 3 Problems and resolutions

EM is laborious and time-consuming and has the following limitations that we should recognize and try to overcome. (i) Samples with low viral particle concentrations ($<10^5$ $L^{-1}$) could lead to low sensitivity of EM. Immunoaggregation with specific antibody or nonimmunological procedures, including ultracentrifugation and agar diffusion, may be used to concentrate the material and therefore yield higher sensitivity [8]. (ii) It can produce artifacts or false-negative results. UA acts as a fixative as well as a stain, and viruses can be viewed as intact long after initial diagnosis. Although PTA actively degrades some viruses, and immediate visualization is possible, viruses degrade within a few days after staining. (iii) Strict experimental conditions are required. If samples are not correctly shipped, preserved or fixed, distortion of viruses and cellular ultrastructure would happen. (iv) The complex embedding procedure is time-consuming. Rapid methods for preparation of sections within 2.5–3 h have been reported. These involve very thin sectioning of tissue blocks (0.5 mm), which allows the length of time in solution to be decreased, and incubation is carried out at higher temperatures for shorter periods [17,84]. The advent of microwave processing has further reduced the time required for staining, dehydration and embedding [85,86]. Combination of microwave technology with digital image acquisition allows one-day diagnosis based on ultrathin sections and worldwide sharing of crucial or interesting findings using modern telemicroscopy tools via the internet [22]. (v) Real-time and live observations may be required for some experiments or investigations. (vi) If the pathology is focal, a small biopsy specimen or a single EM block may miss the area of infection. In this case, the combination of confocal microscopy and EM is recommended. It is important to examine semi-thin sections by light microscopy or to examine large (1–2 cm) sections of wet tissue by confocal microscopy to select areas of abnormal tissue morphology for subsequent EM examination. (vii) EM is unsuitable as a high-through-put screening method. Therefore, digital data analysis systems are required to improve sample and specimen throughput. Semi-automated analysis of digital images based on bispectral features has been described for identifying viruses in negative stains [87].

A combination of EM, IEM and clinical data is recommended to improve the accuracy of functional EM diagnosis and to ameliorate the limitations associated with this technique. Alternatively, Cryo-EM is a powerful tool for investigation of ultrastructural features [88,89]. This technique allows examination of flash-frozen viral suspensions without chemical alteration of structures using a special transmission electron microscope equipped with a cryo-stage and cold chamber.

Reconstruction and analysis software provide a powerful tool for further use of digital data from cryo-EM. Many digital images are acquired at different tilt angles by cryo-EM and reconstructed into a 3D electronic representation [90–94], which permits coloration of different components. Any trace of change in morphology due to mutation or drug treatment can then be detected by comparison with the control group [95]. Therefore, cryo-EM and tomography may provide a useful database of virus and host cell information, including the 3D structure of non-chemically fixed viruses, cell receptors for virus binding and the subunits of naked or enveloped viruses. Such details may provide useful data for drug design and gene therapy research [96–100].

## 4 Conclusion

EM is a fundamental tool in clinical virus diagnosis and research into basic viral mechanisms, particularly in cases where agents are unknown or unsuspected. EM is used for etiological identification in outbreaks with emerging viruses, analysis of morphology and morphogenesis of viral pathogens, and ultrastructural comparisons for anti-viral drug design [101] in gene therapy. It is essential that EM is based on clinical data and comprehensive pathogenesis data from routine histology. Combined use of EM, IEM and cryo-EM [48,49] is recommended to exploit fully the advantages of this technique while minimizing its limitations, although cryo-EM is not a suitable tool for pathogen detection.

*This work was supported by the Major Scientific and Technological Special Project of China (2009ZX10004-216), the National Natural Science Foundation of China (81100809, 81271417) and Fundamental Research Funds for the Central University of China (2011JBM124).*

1 Palese P, Wang T T. H5N1 influenza viruses: facts, not fear. Proc Natl Acad Sci USA, 2012, 109: 2211–2213
2 Woo P C, Lau S K, Wong B H, et al. Feline morbillivirus, a previously undescribed paramyxovirus associated with tubulointerstitial nephritis in domestic cats. Proc Natl Acad Sci USA, 2012, 109: 5435–5440
3 Biel S S, Gelderblom H R. Diagnostic electron microscopy is still a timely and rewarding method. J Clin Virol, 1999, 13: 105–119
4 Biel S S, Gelderblom H R. Electron microscopy of viruses. In: Virus Culture—A Practical Approach. New York: Oxford University Press, 1999. 111–147
5 Goldsmith C S, Miller S E. Modern uses of electron microscopy for detection of viruses. Clin Microbiol Rev, 2009, 22: 552–563
6 Peiris J S, Lai S T, Poon L L, et al. Coronavirus as a possible cause of severe acute respiratory syndrome. Lancet, 2003, 361: 1319–1325
7 Song J D, Qu J G, Lu Z Z, et al. Methods and applications in improving virus detection sensitivity by TEM with negative staining. Bing Du Xue Bao, 2010, 26: 410–413
8 Hazelton P R, Gelderblom H R. Electron microscopy for rapid diagnosis of infectious agents in emergent situations. Emerg Infect Dis, 2003, 9: 294–303
9 Nzounza P, Chazal M, Guedj C, et al. The scaffolding protein Dlg1 is a negative regulator of cell-free virus infectivity but not of cell-to-cell HIV-1 transmission in T cells. PLoS ONE, 2012, 7: e30130
10 Fontana J, Cardone G, Heymann J B, et al. Structural changes in Influenza virus at low pH characterized by cryo-electron tomography. J Virol, 2012, 86: 2919–2929
11 Flewett T H, Beards G M, Brown D W, et al. The diagnostic gap in diarrhoeal aetiology. Ciba Found Symp, 1987, 128: 238–249
12 Kausche G, Pfankuch E, Ruska H. Die Sichtbarmachung von pflanzlichem Virus im Ubermikroskop. Naturwissenschaften, 1939, 27: 292–299
13 Hung T, Yao J E, Li W Z, et al. Ultrastructure and Electron Microscopy in Biological Medicine. Beijing: Science Press, 1980
14 Knipe D M, Howley P M, Griffin D E, et al. Fields' Virology. 5th ed. Philadelphia: Lippincott Williams & Wilkins (a Wolters Kluwer Business), 2007
15 Hung T. Atlas of Hemorrhagic Fever with Renal Syndrome. Beijing: Science Press, 1988
16 Monastyrska I, Ulasli M, Rottier P J, et al. An autophagy-independent role for LC3 in equine arteritis virus replication. Autophagy, 2013, 9: 164–174
17 Doane F W. Virus morphology as an aid for rapid diagnosis. Yale J Biol Med, 1980, 53: 19–25
18 Biel S S, Nitsche A, Kurth A, et al. Detection of human polyomaviruses in urine from bone marrow transplant patients: comparison of electron microscopy with PCR. Clin Chem, 2004, 50: 306–312
19 Hartjen P, Frerk S, Hauber I, et al. Assessment of the range of the HIV-1 infectivity enhancing effect of individual human semen specimen and the range of inhibition by EGCG. AIDS Res Ther, 2012, 9: 2
20 Hung T, Xia S M, Song G, et al. Viruses of classical and mild forms of haemorrhagic fever with renal syndrome isolated in China have similar bunyavirus-like morphology. Lancet, 1983, 1: 589–591
21 Regenmortel M H V van, Bishop D H L, Carstens E B, et al. Virus taxonomy: classification and nomenclature of viruses. In: 7th Report of the International Committee on Taxonomy of Viruses. San Diego: Academic Press, 2000
22 Schroeder J A, Gelderblom H R, Hauroeder B, et al. Microwave-assisted tissue processing for same-day EM-diagnosis of potential bioterrorism and clinical samples. Micron, 2006, 37: 577–590
23 Anson M L, Stanley W M. Some effects of iodine and other reagents on the structure and activity of Tobacco mosaic virus. J Gen Physiol, 1941, 24: 679–690
24 Mudd S, Polevitzky K, Anderson T F, et al. Bacterial morphology as shown by the electron microscope: II. The bacterial cell-wall in the genus *Bacillus*. J Bacteriol, 1941, 42: 251–264
25 Almeida J D, Waterson A P. Some implications of a morphologically oriented classification of viruses. Arch Gesamte Virusforsch, 1970, 32: 66–72
26 Hung T, Xia S M, Zhao T X, et al. Morphological evidence for identifying the viruses of hemorrhagic fever with renal syndrome as candidate members of the Bunyaviridae family. Arch Virol, 1983, 78: 137–44
27 Nagler F P, Rake G. The use of the electron microscope in diagnosis of variola, vaccinia, and varicella. J Bacteriol, 1948, 55: 45–51
28 Welch A B. Purification, morphology and partial characterization of a reovirus-like agent associated with neonatal calf diarrhea. Can J Comp Med, 1971, 35: 195–202
29 Reagan R L, Brueckner A L. Morphological observations by electron microscopy of the Lansing strain of poliomyelitis virus after propagation in the Swiss albino mouse. Tex Rep Biol Med, 1952, 10: 425–428
30 Gust I D, Kaldor J, Cross G F, et al. Virus-like particles associated with a faecal antigen from hepatitis patients and with Australia antigen. Aust J Exp Biol Med Sci, 1971, 49: 1–9
31 Kapikian A Z, Wyatt R G, Dolin R, et al. Visualization by immune electron microscopy of a 27-nm particle associated with acute infectious nonbacterial gastroenteritis. J Virol, 1972, 10: 1075–1081
32 Melnick J L, Phillips C A. Enteroviruses: vaccines, epidemiology, diagnosis, classification. CRC Crit Rev Clin Lab Sci, 1970, 1: 87-118
33 Dane D S, Cameron C H, Briggs M. Virus-like particles in serum of patients with Australia-antigen-associated hepatitis. Lancet, 1970, 1: 695–698
34 Feinstone S M, Kapikian A Z, Purceli R H. Hepatitis A: detection by immune electron microscopy of a viruslike antigen associated with acute illness. Science, 1973, 182: 1026–1028
35 Bishop R F, Davidson G P, Holmes I H, et al. Virus particles in epithelial cells of duodenal mucosa from children with acute non-bacterial gastroenteritis. Lancet, 1973, 2: 1281–1283
36 Johnson K M, Lange J V, Webb P A, et al. Isolation and partial characterisation of a new virus causing acute haemorrhagic fever in Zaire. Lancet, 1977, 1: 569–571
37 Hung T, Chen G M, Wang C G, et al. Rotavirus-like agent in adult non-bacterial diarrhoea in China. Lancet, 1983, 2: 1078–1079
38 Goldsmith C S, Elliott L H, Peters C J, et al. Ultrastructural characteristics of Sin Nombre virus, causative agent of hantavirus pulmonary syndrome. Arch Virol, 1995, 140: 2107–2122
39 Zaki S R, Greer P W, Coffield L M, et al. Hantavirus pulmonary syndrome. Pathogenesis of an emerging infectious disease. Am J Pathol, 1995, 146: 552–579
40 Hyatt A D, Selleck P W. Ultrastructure of equine morbillivirus. Virus Res, 1996, 43: 1–15
41 Hyatt A D, Zaki S R, Goldsmith C S, et al. Ultrastructure of Hendra virus and Nipah virus within cultured cells and host animals. Microbes Infect, 2001, 3: 297–306
42 Nicholls J M, Poon L L, Lee K C, et al. Lung pathology of fatal severe acute respiratory syndrome. Lancet, 2003, 361: 1773–1778
43 Bayer-Garner I B. Monkeypox virus: histologic, immunohistochemical and electron-microscopic findings. J Cutan Pathol, 2005, 32: 28–34
44 Hung T, Chen G M, Wang C G, et al. Waterborne outbreak of rotavirus diarrhoea in adults in China caused by a novel rotavirus. Lancet, 1984, 1: 1139–1142
45 Wong A H, Cheng P K, Lai M Y, et al. Virulence potential of fusogenic orthoreoviruses. Emerg Infect Dis, 2012, 18: 944–948
46 Limonta D, Falcon V, Torres G, et al. Dengue virus identification by transmission electron microscopy and molecular methods in fatal dengue hemorrhagic fever. Infection, 2012, 40: 689–694
47 Wanat K A, Holler P D, Dentchev T, et al. Viral-associated trichodysplasia: characterization of a novel polyomavirus infection with therapeutic insights. Arch Dermatol, 2012, 148: 219–223
48 Rossmann M G. Crystallography, evolution, and the structure of viruses. J Biol Chem, 2012, 287: 9552–9559
49 Beniac D R, Melito P L, Devarennes S L, et al. The organisation of Ebola virus reveals a capacity for extensive, modular polyploidy. PLoS ONE, 2012, 7: e29608
50 Carter S D, Surtees R, Walter C T, et al. Structure, function, and evolution of the Crimean-Congo hemorrhagic fever virus nucleocapsid protein. J Virol, 2012, 86: 10914–10923
51 Van Rooyen C E, Scott G D. Smallpox diagnosis with special reference to electron microscopy. Can J Public Health, 1948, 39: 467–477
52 Chinese Academy of Medical Science. Atlas of Electron Micrographs for Medical Biologic. 5th ed. Beijing: Science Press, 1978
53 Chen H, Williams H N. Sharing of prey: coinfection of a bacterium by a virus and a prokaryotic predator. MBio, 2012, 3: e00051-12
54 Prusiner S B. Prion Biology and Diseases. New York: Cold Spring Harbor Monograph Series 41, 2004
55 Prusiner S B. Prions. Proc Natl Acad Sci USA, 1998, 95: 13363–13383
56 Zhang Y, He J S, Wang X, et al. Administration of amyloid-beta42 oligomer-specific monoclonal antibody improved memory performance in SAMP8 mice. J Alzheimers Dis, 2011, 23: 551–561
57 He J, Zhang Y, Hong T. Progress in the development of therapeutic antibodies targeting prion proteins and beta-amyloid peptides. Sci China Life Sci, 2010, 53: 959–963
58 Zhang Y, Wang X, He J S, et al. Preparation and characterization of a monoclonal antibody with high affinity for soluble Abeta oligomers. Hybridoma (Larchmt), 2009, 28: 349–354
59 Isas J M, Luibl V, Johnson L V, et al. Soluble and mature amyloid fibrils in drusen deposits. Invest Ophthalmol Vis Sci, 2010, 51: 1304–1310
60 Jones P H, Mehta H V, Maric M, et al. Bone marrow stromal cell antigen 2 (BST-2) restricts mouse mammary tumor virus (MMTV) replication *in vivo*. Retrovirology, 2012, 9: 10
61 Hansman G S, Taylor D W, McLellan J S, et al. Structural basis for broad detection of genogroup II noroviruses by a monoclonal antibody that binds to a site occluded in the viral particle. J Virol, 2012, 86: 3635–3646
62 Sanchez E G, Quintas A, Perez-Nunez D, et al. African swine fever virus uses macropinocytosis to enter host cells. PLoS Pathog, 2012, 8: e1002754
63 Schelhaas M, Shah B, Holzer M, et al. Entry of human papillomavirus type 16 by actin-dependent, clathrin- and lipid raft-independent endocytosis. PLoS Pathog, 2012, 8: e1002657
64 Patterson S, Russell W C. Ultrastructural and immunofluorescence studies of early events in adenovirus-HeLa cell interactions. J Gen Virol, 1983, 64: 1091–1099
65 Liu Z, Liu S, Cui J, et al. Transmission electron microscopy studies of cellular responses to entry of virions: one kind of natural nanobiomaterial. Int J Cell Biol, 2012, 2012: 596589
66 Stoneham C A, Hollinshead M, Hajitou A. Clathrin-mediated endocytosis and subsequent endo-lysosomal trafficking of adeno-associated virus/phage. J Biol Chem, 2012, 287: 35849–35859
67 Romero-Brey I, Merz A, Chiramel A, et al. Three-dimensional architecture and biogenesis of membrane structures associated with hepatitis C virus replication. PLoS Pathog, 2012, 8: e1003056
68 Ooms L S, Jerome W G, Dermody T S, et al. Reovirus replication protein mu2 influences cell tropism by promoting particle assembly within viral inclusions. J Virol, 2012, 86: 10979–10987
69 Kolesnikova L, Heck S, Matrosovich T, et al. Influenza virus budding from the tips of cellular microvilli in differentiated human airway epithelial cells. J Gen Virol, 2013 (in press)
70 Houzet L, Gay B, Morichaud, et al. Intracellular assembly and budding of the Murine Leukemia Virus in infected cells. Retrovirology, 2006, 3: 12
71 Schubert U, Ott D E, Chertova E N, et al. Proteasome inhibition interferes with gag polyprotein processing, release, and maturation of HIV-1 and HIV-2. Proc Natl Acad Sci USA, 2000, 97: 13057–13062
72 Huang C Y, Chiang S F, Lin T Y, et al. HIV-1 Vpr triggers mitochondrial destruction by impairing Mfn2-mediated ER-mitochondria interaction. PLoS ONE, 2012, 7: e33657
73 Boyapalle S, Wong T, Garay J, et al. Respiratory syncytial virus NS1 protein colocalizes with mitochondrial antiviral signaling protein MAVS following infection. PLoS ONE, 2012, 7: e29386
74 Le Sage V, Banfield B W. Dysregulation of autophagy in murine fibroblasts resistant to HSV-1 infection. PLoS ONE, 2012, 7: e42636
75 Lam S, Chen K C, Ng M M, et al. Expression of plasmid-based shRNA against the E1 and nsP1 genes effectively silenced Chikungunya virus replication. PLoS ONE, 2012, 7: e46396
76 Alfonso V, Maroniche G A, Reca S R, et al. AcMNPV core gene ac109 is required for budded virion transport to the nucleus and for occlusion of viral progeny. PLoS ONE, 2012, 7: e46146
77 Nagel C H, Dohner K, Binz A, et al. Improper tagging of the non-essential small capsid protein VP26 impairs nuclear capsid egress of Herpes simplex virus. PLoS ONE, 2012, 7: e44177
78 Hung T, Zhou J Y, Tang Y M, et al. Identification of Hantaan virus-related structures in kidneys of cadavers with haemorrhagic fever with renal syndrome. Arch Virol, 1992, 122: 187–199
79 Friedlaender M, Moore D H, Koprowski H. Studies with the electron microscope of virus-host relationships in Ehrlich ascites tumor cells. II. The localization and possible development of anopheles A virus within the endoplasmic reticulum of the host cell. J Exp Med, 1955, 102: 371–378
80 Friedlaender M, Moore D H, Love R, et al. Studies with the electron microscope of virus-host relationships in Ehrlich ascites tumor cells. I. The identification and structure of anopheles A virus. J Exp Med, 1955, 102: 361–370
81 Ke P Y, Chen S S. Activation of the unfolded protein response and autophagy after hepatitis C virus infection suppresses innate antiviral immunity *in vitro*. J Clin Invest, 2011, 121: 37–56
82 Geisbert T W, Jahrling P B. Differentiation of filoviruses by electron microscopy. Virus Res, 1995, 39: 129–150
83 Gasper-Smith N, Crossman D M, Whitesides J F, et al. Induction of plasma (TRAIL), TNFR-2, Fas ligand, and plasma microparticles after human immunodeficiency virus type 1 (HIV-1) transmission: implications for HIV-1 vaccine design. J Virol, 2008, 82: 7700–7710
84 Doane F W, Anderson N, Chao J, et al. Two-hour embedding procedure for intracellular detection of viruses by electron microscopy. Appl Microbiol, 1974, 27: 407–410
85 Visser C E, Voute A B, Oosting J, et al. Microwave irradiation and cross-linking of collagen. Biomaterials, 1992, 13: 34–37
86 Webster P. Microwave-assisted processing and embedding for transmission electron microscopy. Methods Mol Biol, 2007, 369: 47–65
87 Ong H, Chandran V. Identification of gastroenteric viruses by electron microscopy using higher order spectral features. J Clin Virol, 2005, 34: 195–206
88 Zhang R, Hryc C F, Cong Y, et al. 4.4 A cryo-EM structure of an enveloped alphavirus Venezuelan equine encephalitis virus. EMBO J, 2011, 30: 3854–3863
89 Malet H, Canellas F, Sawa J, et al. Newly folded substrates inside the molecular cage of the HtrA chaperone DegQ. Nat Struct Mol Biol, 2012, 19: 152–157
90 Liu J, Bartesaghi A, Borgnia M J, et al. Molecular architecture of native HIV-1 gp120 trimers. Nature, 2008, 455: 109–113
91 Provencher S W, Vogel R H. Three-dimensional reconstruction from electron micrographs of disordered specimens. I. Method. Ultramicroscopy, 1988, 25: 209–221
92 Sougrat R, Bartesaghi A, Lifson J D, et al. Electron tomography of the contact between T cells and SIV/HIV-1: implications for viral entry. PLoS Pathog, 2007, 3: e63
93 Subramaniam S, Bartesaghi A, Liu J, et al. Electron tomography of viruses. Curr Opin Struct Biol, 2007, 17: 596–602
94 Vogel R H, Provencher S W. Three-dimensional reconstruction from electron micrographs of disordered specimens. II. Implementation and results. Ultramicroscopy, 1988, 25: 223–239
95 Ryner M, Stromberg J O, Soderberg-Naucler C, et al. Identification and classification of human cytomegalovirus capsids in textured electron micrographs using deformed template matching. Virol J, 2006, 3: 57
96 Taube S, Rubin J R, Katpally U, et al. High-resolution X-ray structure and functional analysis of the murine norovirus 1 capsid protein protruding domain. J Virol, 2010, 84: 5695–5705
97 Carnall J M, Waudby C A, Belenguer A M, et al. Mechanosensitive self-replication driven by self-organization. Science, 2010, 327: 1502–1506
98 Morgan G W, Hollinshead M, Ferguson B J, et al. Vaccinia protein F12 has structural similarity to kinesin light chain and contains a motor binding motif required for virion export. PLoS Pathog, 2010, 6: e1000785
99 Cong Y, Baker M L, Jakana J, et al. 4.0-A resolution cryo-EM structure of the mammalian chaperonin TRiC/CCT reveals its unique subunit arrangement. Proc Natl Acad Sci USA, 2010, 107: 4967–4972
100 Levy H C, Bostina M, Filman D J, et al. Catching a virus in the act of RNA release: a novel poliovirus uncoating intermediate characterized by cryo-electron microscopy. J Virol, 2010, 84: 4426–4441
101 Austin S K, Dowd K A, Shrestha B, et al. Structural basis of differential neutralization of DENV-1 genotypes by an antibody that recognizes a cryptic epitope. PLoS Pathog, 2012, 8: e1002930

**Open Access** This article is distributed under the terms of the Creative Commons Attribution License which permits any use, distribution, and reproduction in any medium, provided the original author(s) and source are credited.
